# COVID-19 vaccine hesitancy and short-term and long-term intentions among unvaccinated young adults: a mixed-method approach

**DOI:** 10.1186/s12889-022-14448-3

**Published:** 2022-11-07

**Authors:** Soojung Kim, Erin Willis, Shane Wehlage, Hannah Scheffer-Wentz, Michael Dulitz

**Affiliations:** 1 Department of Communication, University of North Dakota, O’Kelly Hall Room 214, 221 Centennial Dr. Stop 7169, Grand Forks, ND 58202-7169 USA; 2 Advertising, Public Relations & Media Design, University of Colorado Boulder, Boulder, CO 80309 USA; 3 Opioid Response Project Coordinator and COVID-19 Data and Analytics Leader, Grand Forks Public Health, 151 S 4th Street Suite N301, Grand Forks, ND 58201 USA

**Keywords:** COVID-19, Vaccine hesitancy, Vaccination intention, Health belief model

## Abstract

**Background:**

Only 63.8% of Americans who are 18-to-24-years-old have been fully vaccinated for COVID-19 as of June 1, 2022. The Grand Forks County, North Dakota is facing a similar challenge. As of June 2022, 47% of individuals in the 19-to-29-year-old age group are vaccinated. Focusing on unvaccinated individuals in their 20s, Study 1 aims to understand the ways in which receiving COVID-19 vaccines is construed using qualitative interviews; and Study 2 compares the predictors of short-term vaccination intention (i.e., next month) with those of long-term vaccination intention (i.e., three to 5 years) using an online survey.

**Methods:**

For Study 1, we conducted five focus groups and four in-depth interviews via Zoom with a total of 26 unvaccinated individuals in their 20s living in the Grand Forks County. Constant comparison process was used to categorize data into themes and to recognize characteristics of the identified themes. The aim was to develop themes and associated characteristics. For Study 2, we conducted an online survey with a convenience sample of 526 unvaccinated individuals. Logistic regression estimated odds ratios (OR) and 95% confidence intervals (CI) for associations between attitudes, perceptions, and beliefs in misinformation and short-term and long-term vaccination intentions, accounting for demographics and socioeconomic status.

**Results:**

In Study 1, two themes were identified: feelings of uncertainty sparked by profits and monetization and navigating the fear of the unknown. In Study 2, an increase in the confidence of COVID-19 vaccines showed significantly higher odds of short-term intention (OR = 2.658, 95%CI 1.770, 3.990) and long-term intention (OR = 1.568, 95% CI 1.105, 2.226). Believing in misinformation had significantly lower odds of short-term intention (OR = 0.712, 95%CI 0.513, 0.990), while more positive attitudes (OR = 1.439, 95% CI 1.024, 2.024), stronger preference in calculating the benefits of COVID-19 vaccines (OR = 2.108, 95% CI 1.541, 2.882), and greater perceived susceptibility (OR = 1.471, 95% CI 1.045, 2.070) to and severity of contracting COVID-19 (OR = 1.362, 95% CI 1.020, 1.820) were significantly associated with higher odds of long-term intention.

**Conclusions:**

Short-term and long-term intentions were predicted differently. Instilling strong confidence in COVID-19 vaccines should increase both short-term and long-term intentions.

## Background

The World Health Organization (WHO) was first notified of a group of pneumonia cases in Wuhan City, China in December 2019 [1]. This was later identified as COVID-19, an acute respiratory syndrome, caused by the SARS-CoV-2 virus. COVID-19 began to spread around the world; the first positive case in the U.S. was reported in January 2020 [2]. Since then, there have been 85.1 million confirmed cases and 1.01 million deaths related to COVID-19 in the U.S. alone [3]. A speedy vaccine development and administration campaigns allowed 590 million doses of COVID-19 vaccines to be administered, resulting in 222 million fully vaccinated Americans. Worldwide, 11.7 billion COVID-19 vaccinations have been administered, helping 4.66 billion individuals to be fully vaccinated [4]. Ample evidence shows how the COVID-19 vaccination has lowered hospitalization and mortality rates [5–9].

Nonetheless, the U.S. has hit a vaccine-enthusiasm plateau at the national level [10, 11]. Some vaccine-hesitancy might be blamed on misinformation and/or disinformation. Misinformation is incorrect or incomplete facts, whereas disinformation is false information; during the global pandemic, an “infodemic” proliferated across the U.S., and social media facilitated the spread of mis- and disinformation [12–14]. There are still many Americans who are not yet vaccinated against COVID-19 [15]. Specifically, only 63.8% of Americans who are 18-to-24-years-old have been fully vaccinated as of June 1, 2022 [16]. Young adults tend to delay receiving COVID-19 vaccines despite the wide availability of vaccines [17]. This phenomenon is called vaccine hesitancy [18, 19]. Vaccine hesitancy in young adults results potentially from experiencing only mild symptoms after COVID-19 infection and/or their relatively lower risks of hospitalization and morality compared to older adults [5, 20].

The Grand Forks County in North Dakota is facing a similar challenge on how to improve the community’s COVID-19 vaccination rates in hopes of achieving herd immunity. As of June 2022, only 47% of individuals in the 19-to-29-year-old age group are fully vaccinated [21]. This makes them the largest group of unvaccinated individuals in the community, in which approximately 25% of county population (66,861) [22] account for the 20-to-29-year-old age group [23]. Notably, 20% of individuals in the 19-to-29-year-old age group were fully vaccinated as of April 2021 and 45% as of September 2021 [21]. In other words, it took nine months since September 2021 to reach the 47% level in June 2022, mirroring the plateau at the national level [10, 11]. The State of North Dakota and peer counties in the state, such as Ward, Cass, and Burleigh, face the same battle against low vaccination rates and plateaus in young populations.

Scientists and experts forecast that the likelihood of the world “living” with COVID-19 for a longer period of time is high [24, 25]. Some anticipate that COVID-19 will resemble the seasonal flu [26, 27]. The efforts to increase vaccine uptake started as a sprint: the sooner everyone is vaccinated, the sooner we can “end” the pandemic [28]. Yet, evidence is increasingly pointing in the direction that vaccine promotion efforts moving forward will be a marathon [24]. In this light, a different approach to understanding and addressing COVID-19 vaccine hesitancy among those who have yet to be vaccinated is needed. Therefore, this study applied the Health Belief Model (HBM) and psychological antecedents of vaccination (5C) as they provide comprehensive theoretical frameworks to understand factors contributing to COVID-19 vaccine hesitancy. The HBM is a cognitive model that helps to explain why people engage in health behaviors [29]. The constructs of HBM (i.e., perceived susceptibility, perceived severity, perceived benefits, perceived barriers, cues to action, and self-efficacy) try to identify key decision-making points that influence health behaviors. In addition, three of the five psychological antecedents of vaccination (5C) (i.e., confidence, calculation, and collective responsibility) are particularly relevant to COVID-19 vaccination [30, 31].

Focusing on the Grand Forks County in North Dakota, the first objective of this research is to understand the ways in which receiving COVID-19 vaccines is construed among unvaccinated individuals who are 19-to-29-years-old using qualitative interviews (Study 1). Second, considering a much-needed long-term perspective, this research hopes to compare the predictors of short-term vaccination intention (i.e., next month) with those of long-term vaccination intention (i.e., three to five years) using an online survey among unvaccinated individuals in an effort to inform better health promotion strategies (Study 2). The HBM and 5C guided the construction of the survey to better understand what beliefs this age group holds about vaccination and to identify barriers to action. Given that younger age [32–34] and living in the Midwest [35] are associated with greater vaccine hesitancy, this regional snapshot could contribute to addressing the challenges of COVID-19 vaccine promotion that public health professionals face. Moreover, a mixed-method approach involving both qualitative interview and quantitative survey methods has rarely been used together to understand unvaccinated adults’ COVID-19 vaccine hesitancy, except for a few studies that recruited healthcare workers [36]. Doing so does not only advance the literatures of vaccine hesitancy; but it also provides a rich picture of COVID-19 vaccine hesitancy for public health professionals to develop effective messaging and interventions targeted to “vaccine non-adopters” [37] particularly via social media.

## Study 1 Methods

### Sample

Participants were recruited through a social media promotion by the Grand Forks Public Health Department, and all met the eligibility criteria in that they: (1) had home addresses in the Grand Forks County, North Dakota; (2) had never been vaccinated against COVID-19; and (3) were in the 19-to-29-year-old age group. Participants joined either a focus group session or an in-depth interview via Zoom depending on their preference and schedule. All participants received a $30 gift card upon the completion of the interview.

### Procedure

Focus groups and in-depth interviews were conducted from February 2022 to March 2022. Once the participants entered the Zoom session, researchers greeted participants and introduced themselves first. Informed consent was obtained verbally, followed by participants’ introductions. Researchers then asked a series of questions, asking their opinions and attitudes toward COVID-19 vaccines. Each focus group session took approximately an hour to 90 minutes, and each in-depth interview session took approximately 30 to 40 minutes. All Zoom sessions were recorded and later transcribed. The IRB approval was obtained from the University of North Dakota, which considered it “expedited” (IRB # IRB0004426).

## Study 1 Results

This study identified two themes: feelings of uncertainty sparked by profits and monetization and navigating the fear of the unknown. Each theme is described below in more detail.

### Feelings of uncertainty sparked by profits and monetization

Participants perceived there to be financial interest by the Centers for Disease Control and Prevention (CDC) and by the pharmaceutical drug companies involved in making the COVID-19 vaccines, such as Pfizer and Moderna. Much of the communication to the public addressed the different vaccine brands available and the number of shots required. Since the vaccines were available to the public, free of charge or with a financial incentive, some participants felt pharmaceutical companies and government organizations were profiting off the pandemic. Most of the participants’ skepticism was rooted in distrust of government and big business. Additionally, because the vaccines did not follow the traditional Food and Drug Administration (FDA)’s approval process, many participants questioned its safety and efficacy.

“I think the vaccine, some people are using it as a business … people are using vaccines to gain some money or something.” [Focus group #5, Participant 23]

“I don’t trust like big pharmacy companies because I think there’s a lot of money involved with this, for sure. Like for funding the vaccine or money received from the vaccines, so I don’t necessarily trust them.” [Focus group #2, Participant 6]

“The vaccine is just untrustworthy. Like, I don’t know, I don’t trust it yet at all. My concern about the COVID vaccine is the rapid rate at which it came out.” [Focus group #3, Participant 11]

Due to the lack of communication about the FDA’s emergency use authorization and the implications for the approval process, many questioned the intentions of both pharmaceutical companies and the government. Some participants questioned the COVID-19 vaccine because of the trial process and the classification of the FDA’s emergency approval. Other participants felt as if research related to the COVID-19 vaccine was scant and needed to be expanded before the vaccine could be deemed safe.

“There is still a lot of research that needs to be done in order to determine the safety and effectiveness of them.” [Focus group #4, participant 17]

“It’s my understanding that there’s some vaccines that have been through like a 7-year trial. So, I’d be more comfortable with having like a 7- to 10-year trial. I know that’s a long time, but if we’re going to create a vaccine and produce it and actually like push it … I know it’s harder because we’re trying to create a herd immunity, but we don’t know exactly what’s going to happen with that.” [Focus group #4, participant 15]

“I was very interested in how fast they got the vaccine out compared to other vaccines.” [Focus group #2, participant 6]

Participants expressed distrust for government and pharmaceutical companies because they felt as if the situation was being monetized and that people were being tricked into taking the COVID-19 vaccine. Additionally, many local and state governments incentivized getting vaccinated, but this only bred more distrust in the vaccine. Participants said that if the vaccine was effective and safe, there would not be any reason to incentivize people taking it. Participants questioned the government’s strategy in requiring the vaccine for employment or for travel, as an example.

“Why should we be paid to put things into our body when we don’t get paid for anything else?” [Focus group #4, participant 15]

“I feel like I’m the type of person where if somebody is forcing me to do something, it makes me want to do it less and less. So. Yeah. It definitely discourages me with all the mandates that are put into place.” [Focus group #3, participant 11]

Most participants were ambivalent to the vaccine. Few held strong beliefs, either to be or not to be vaccinated. Several participants discussed the politicization of the issue, and how sometimes opinions can be polarizing. Many of the participants did not show great concern for COVID-19, and those who did had survived the virus with mild symptoms and/or knew someone with long-COVID symptoms. Several reported knowing someone who experienced negative side effects from the COVID-19 vaccine. Others did not believe that the vaccine was effective due to breakthrough cases. There was some debate on the purpose of vaccines, and what is reasonable to expect after receiving the COVID-19 vaccine.

“I personally have never thought of being vaccinated because of the side effects of the vaccine that I’ve been hearing of, yeah.” [Focus group #5, participant 24]

“I question getting a vaccine that won’t protect you from getting the virus.” [Focus group #4, participant 19]

“So, I guess I haven’t like put any really hard thought into it. For me, it’s just kind of you get it, or you don’t. I’m not completely against it at all. I’m just not 100% for it. I know I did want to wait, you know, for it to be out longer and see how other people reacted to getting it and what not. But like I said, I’m not really against it, or for it. I’m just kind of neutral right now.” [In-depth interview #2].

Few in the sample spoke with any urgency related to receiving the COVID-19 vaccine. Participants did not trust the different players or the game and felt as if there were too many unknowns to receive the vaccine with confidence.

### Navigating the fear of the unknown

Most of the participants expressed the fear of the unknown. One participant said she was “extremely anxious” about the current landscape amidst the pandemic. Participants feared the future due to the changes they experienced during the economic shut down in 2020 and following. Despite being tired of hearing about COVID-19 and the vaccine, even while feeling ready to “go back to normal,” participants also recognized that the pandemic was not yet over, and more change could still happen. Nonetheless, the unknown was often a factor in this group’s decision-making.

Participants reported keeping up with the changing information landscape, following government reporting, and news from organizations, such as the WHO or the CDC. Information often became the source of anxiety.

“What worries me about this vaccine is the fact that there are so many variations of the same vaccine … well, they all have effects to some extent. But I really don’t see the reason as to why we should have several vaccines for managing one condition.” [Focus group #1, participant 4]

Some participants shared that they heard of side effects from both COVID-19 and the vaccination, thus discouraging them from being vaccinated. These accounts were often shared on social media or through word-of-mouth. Almost all the participants actively looked for health information online, especially on social media. Participants did recognize the risks of online health information, with several saying they look at multiple sources to evaluate content. However, the stories about side effects were enough to deter several participants from the vaccine.

“Yeah, some people experience pains, you know, sharp pains from getting vaccinated, for a few hours, and some it takes days. Not being able to move around, mobility issues and all of that, just from getting vaccinated. Why should I experience that if the vaccine is flawed? I mean, I believe that definitely it’s not guaranteed. I believe there are long-term side effects to it.” [Focus group #1, participant 1]

“You know, the rumors you hear on that aspect of things and I feel like a vaccine should not affect your reproductive system whatsoever, and just the fact that the media put some of that stuff out – it’s just scary … ” [Focus group #2, participant 7]

“Like, it’s overwhelming what is portrayed on social media, and so I kind of had to stop really look into it because it just got into rabbit holes that made me super confused and I never knew what was the truth … ” [Focus group #2, participant 6]

Many participants recognized the presence of conspiracy theories, but also believed different aspects of mistruths, or questioned the facts. Many participants did not denounce conspiracy theories, but instead empowered parts of the mistruths to justify their own position to not receive the COVID-19 vaccine. Participants pointed to mass media and social media for the confusion, noting that these information outlets played a role in their own response to COVID-19. Participants did not feel it should be this difficult to decide whether or not to take the vaccine.

“I’m definitely not like an anti-vaxxer by any means, but I would say I was hesitant about the COVID-19 vaccine. I think a few years back, I had to get my flu shot and then I had to do a tetanus shot too. I was less hesitant. I just did it because it’s been around for a very, very long time, and you know, when they start talking about the mRNA in the COVID vaccine versus the other ones that aren’t in it. I guess that makes me more hesitant.” [Focus group #2, participant 7]

“I think the COVID-19 vaccine is a business because, how can you create a vaccine for a virus in a few years, and yet, some other viruses have been with us for more than 20 years and I’ve never gotten a vaccine. And there are also other conspiracy theories behind it. And it’s really hard for us to understand if the other conspiracy theories are true or maybe they are educative.” [Focus group #1, participant 3]

“A lot of times, it has to do with the tone and how the conversation goes, because if I counteract or counter what they’re saying, and they don’t have a response to me and it seems like it’s just based on what they believe versus what is true. Because yeah, I have my own religious beliefs and beliefs about things, but I also like science and scientific stuff, so if it’s not proven, you know, and it’s barely in a like theoretical phase, why are we saying you have to or this is how it is?” [In-depth interview #4]

Participants discussed the atmosphere of the pandemic and how they navigated the fear of the unknown. With so much changes in the past 2 years, participants all suspected more change in the future. While a few participants were definitely not interested in the COVID-19 vaccine, some expressed that they might decide to receive the vaccine in the future. Participants indicated that time would help; they are hesitant to receive COVID-19 vaccines in the near future, but they might be more willing to do so in the distant future.

For me it would just be like time, in a sense, like if I see in like 5 years that everybody who got vaccination 2 years ago it’s like alive healthy living their best life, then I’d be more likely to get I [Focus group #3, participant 10].

I don’t want to get it until I know what will happen to me in 5, 10 years. [Focus group #3, participant 11].

I feel like it’s just I’m one of those people where it’s like give you know, give them 5 years and yeah, we’ll talk about it. But it’s just like it just barely came out it’s been a year, so I don’t know. [In-depth interview #1].

I’d be more comfortable with having like a seven to ten year and I know that that’s a long time [Focus group #4, participant 15].

I think I’m in a similar, similar time frame, I know, in order to know really long-term effects, that is, you do have to wait a generation or within that fifty to 5 years [Focus group #4, participant 17].

## Study 1 discussion

The findings from Study 1 identified multi-layered barriers among 19-to-29-year-olds to receiving the COVID-19 vaccine. The first barrier to vaccination is not understanding the short and long-term effects of COVID-19 or the vaccine. As participants perceived themselves to be healthy, they felt as if they would survive the virus and would rather take their chances with COVID-19 than with the unknowns of the vaccine. The second barrier is the seemingly contradictory information from multiple stakeholders (e.g., healthcare system, pharmaceutical drug companies, local, state, and federal public health agencies). Over the evolution of the pandemic, science has evolved, thus has COVID-19 information. Participants felt as if the information from stakeholders was conflicting and made them question its accuracy. With science constantly evolving and communication rarely keeping up, there is opportunity for confusion and misunderstanding.

The finding from Study 1 is not meant to be generalized to a wider population or to compare the roles of different barriers due to its qualitative nature. Nonetheless, it is worth noting that several participants in Study 1 differentiated the behavior of receiving a vaccine in the near future from performing the same behavior three to five years later or even 10 years later. Therefore, Study 2 hopes to identify predictors of intentions to receive COVID-19 vaccines by using different time frames, namely, in the next month and in the next three to five years.

## Study 2 methods

### Sample

An a priori G*Power analysis was conducted to determine the minimum sample size needed to test the hypotheses [38]. The analysis indicated that the minimum sample size to achieve 80% power for detecting a medium effect size at an α = .05 was 411 for logistic regression analyses, after controlling for covariates. A convenience sample of 564 participants were recruited from Facebook promotions by the Grand Forks Public Health Department and online banner ads that were targeting individuals who are 18-to-34-years-old and are either renters or homeowners in the Grand Forks County. The principal eligibility criteria were that participants were within the 19-to-29-year-old group, had never been vaccinated against COVID-19, and lived in Grand Forks County, North Dakota. Potential participants were excluded if they were less than 18 years old or older than 29 years old; they had at least one dose of a COVID-19 vaccine; and they lived outside of Grand Forks County, North Dakota. The obtained sample size of 564 was deemed appropriate to test the study hypothesis.

### Procedure

The Grand Forks Public Health Department published a Facebook post promoting the online survey in late March 2022. In addition, working with a Fargo, North Dakota-based advertising agency, Off the Wall Advertising, Inc., online banner ads were created to serve homeowners and renters in Grand Forks County who were 18-to-34-years-olds. Upon clicking the ad, they were directed to the online survey that was built on Qualtrics. The online survey included a geo-location tag where it would only allow individuals who access the survey from the Grand Forks County to complete the survey. Grand Forks County residents who were willing to proceed with the online survey questionnaire were asked to read the consent form first. They were then asked to answer eligibility questions inquiring whether they had never received vs. received at least dose of COVID-19 vaccine; whether they were within the age group of 19 to 29; and whether they lived in the Grand Forks County. The IRB approval was obtained from the University of North Dakota, which considered it “expedited” (IRB # IRB0004426).

### Measurements

Intention to receive COVID-19 vaccines were measured by asking two questions: [39] “How likely would you get a COVID-19 vaccine in the next month?” and “How likely would you get a COVID-19 vaccine in the next 3-5 years?” The response options ranged from 1 (“extremely unlikely”) to 5 (“extremely likely”). These two intention questions were later recoded into dichotomous variables as 0 (“extremely unlikely”, “somewhat unlikely”, and “unsure”) or 1 (“somewhat likely” and “extremely likely”).

Among five psychological antecedents of vaccination (5C), three antecedents of vaccine hesitancy were measured [30]. First, confidence was measured by using three 7-point Likert scales: “I am completely confident that COVID-19 vaccines are safe”; “COVID-19 vaccinations are effective”; and “Regarding COVID-19 vaccines, I am confident that public authorities decide in the best interest of the community.” Second, collective responsibility was assessed by utilizing two 7-point Likert scales: “I would get vaccinated because I can also protect people with a weaker immune system” and “COVID-19 vaccination is a collective action to prevent the spread of diseases.” Third, calculation was assessed by using three 7-point Likert scales: “When I think about getting vaccinated against COVID-19, I weigh benefits and risks to make the best decision possible”; “For COVID-19 vaccination, I closely consider whether it is useful for me”; and “It is important for me to fully understand the topic of COVID-19 vaccination, before I get vaccinated.” The response options ranged from 1 (“strongly disagree”) to 7 (“strongly agree”).

Attitudes toward receiving COVID-19 vaccinations were measured by asking the following four 7-point semantic differential scales. The response options ranged from 1 (“bad”, “negative”, “unfavorable”, and “harmful”) to 7 (“good”, “positive”, “favorable”, and “beneficial”).

Four constructs from the Health Belief Model (HBM) were measured using 7-point Likert scales, ranging from 1 (“strongly disagree”) to 7 (“strongly agree”) [40]. First, perceived susceptibility, which refers to individuals’ evaluation on the degree to which they are susceptible to a disease, was assessed by using the following three statements: “I may get infected with COVID-19 if I do not get COVID-19 vaccines”; “I may be hospitalized due to COVID-19 if I do not get COVID-19 vaccines”; and “I may die from COVID-19 if I do not get COVID-19 vaccines.” Second, perceived severity, which refers to individuals’ evaluation of the severity of a disease, was measured by the following two statements: “Complications from COVID-19 are serious” and “Recovering from COVID-19 would take a long time.” Third, perceived benefits, which refers to beliefs on the effectiveness of a recommended health behavior to protect themselves from a disease, were measured by the following three statements: “The COVID-19 vaccines will reduce my likelihood of infection”; “The COVID-19 vaccines will reduce my likelihood of hospitalization due to COVID-19”; and “The COVID-19 vaccines will reduce my likelihood of death from COVID-19.” Finally, perceived barriers, which refers to the perception of the challenges to performing a recommended health behavior, were measured by utilizing the following three statements “The COVID-19 vaccines have concerning side effects”; “I am doubtful about the efficacy of the COVID-19 vaccines”; and “The COVID-19 vaccines are developed way too fast.”

Misinformation was measured by using the following seven statements: [41] (1) “The COVID-19 vaccine is not safe because it was rapidly developed and tested”; (2) “Individuals who have already had COVID-19 and recovered don’t need to get a COVID-19 vaccine”; (3) “COVID-19 vaccines cause infertility (i.e., unable to have children) or miscarriage”; (4) “The COVID-19 vaccines don’t work because you can still get COVID after vaccination”; (5) “The vaccine will alter my DNA”; (6) “The COVID-19 vaccine is part of a big business plan by pharmaceutical companies”; and (7) “The COVID-19 vaccines don’t work because you can still be hospitalized due to COVID or even die from it.” The responses options ranged from 1 (“definitely false”) to 5 (“definitely true”).

Demographics and socioeconomic status were measured as follows: race (White vs. non-White), household income (high ≥ $50,000 vs low < $50,000), religion (Christians vs. non-Christians), job (clerical vs. all others), gender (males vs. females), ethnicity (non-Hispanics vs. Hispanics), age (continuous), and political ideology (7-point scale ranging from 1 [“extremely liberal”] to 7 [“extremely conservative”]) [42].

### Statistical analysis

First, descriptive analyses were obtained. Logistic regression models then estimated odds ratios (OR) and 95% confidence intervals (CI) for the association between independent variables and two outcome variables: intention to receive COVID-19 vaccinations in the next month and in the next three to five years. Models included participants’ demographic and socioeconomic factors, namely, race, household income, religion, job, gender, ethnicity, age, and political ideology. Next, eight variables were included in the model including, attitudes toward receiving COVID-19 vaccinations, confidence, collective responsibility, calculation, perceived severity, perceived susceptibility, perceived benefits, and perceived barriers. Fully adjusted models included seven misinformation variables. All analyses were completed with SPSS version 28.

## Study 2 results

Participants began enrolling in March 2022 with 564 potential participants. Twenty-two were ineligible because they reported that they had received at least one dose of COVID-19 vaccination, and additional 14 participants refused to answer this question, becoming ineligible to proceed. Moreover, two participants became ineligible because they indicated that they did not live in the Grand Forks County. Consequently, the final data analyses included a total of 526 participants.

### Scale construction

Three antecedents of vaccine hesitancy were created by averaging three Likert scales for confidence, two Likert scales for collective responsibility, and three Likert scales for calculation. Reliability statistics among measurement scales for confidence (Cronbach’s α = .79), collective responsibility (Spearman-Brown coefficient = .71), and calculation (Cronbach’s α = .80) were found to be acceptable. Attitudes toward receiving COVID-19 vaccinations variable was created by averaging four semantic differential scales, and reliability statistics among measurement scales were excellent (Cronbach’s α = .86). Perceived susceptibility (Cronbach’s α = .70), perceived benefits (Cronbach’s α = .70), and perceived barriers (Cronbach’s α = .70) were constructed by averaging three Likert scales, and perceived severity (Spearman-Brown coefficient = .70) were created by averaging two Likert scales based on acceptable reliability statistics among measurement scales.

### Participants’ characteristics

The average age was 25.04 years, ranging from 18 to 29 (SD = 2.08). A total of 337 participants (68.5%) were male and 155 (31.5%) were female. Most self-identified as non-Hispanic (*n* = 363, 73.6%) and White (*n* = 360, 73.2%). Approximately half of participants reported less than $50,000 as their total household income (*n* = 255) and identified as Christians (*n* = 247). In terms of job, clerical, office, or sales (e.g., secretary, receptionist, or sales clerk) were the most popular type of jobs held by participants (*n* = 225, 46.2%), followed by manager, executive, or official (e.g., store manager, business executive) (*n* = 80, 16.4%) and service work (e.g., waiter/waitress, hairstylist, police or fireman, janitor, nurses’ aid) (*n* = 76, 15.6%). The majority of participants (*n* = 389, 80.3%) considered themselves liberal. The characteristics of participants are shown in Table 1.

**Table 1:** Characteristics of participants

	N	%
**Sex**		
Female	155	31.5
Male	337	68.5
**Ethnicity**		
Hispanic	130	26.4
Non-Hispanic	363	73.6
**Race**		
White	360	73.2
Non-White	132	26.8
**Household income**		
< $50,000	255	51.8
≥ $50,000	237	48.2
**Religion**		
Christian	247	50.2
Non-Christian	245	49.8
**Job**		
Clerical/Office/Sales	225	46.2
Others	262	53.8
**Political ideology**		
Extremely liberal	96	19.8
Moderately liberal	204	42.1
Slightly liberal	89	18.4
Neither liberal nor conservative	50	10.3
Slightly conservative	27	5.6
Moderately conservative	19	3.9
Extremely conservative	0	0

### Predicting intention to receive COVID-19 vaccines

For individuals’ intention to receive COVID-19 vaccines in the next month, compared to non-whites and non-Christians, whites (OR = 0.296, 95%CI 0.140, 0.627) and Christians (OR = 0.410, 95%CI 0.220, 0.767) had significantly lower odds of intention to get vaccinated in the next month, respectively (Model 3, Table 2). Furthermore, an increase in confidence in COVID-19 vaccines showed significantly higher odds of intention to get vaccinated in the next month (OR = 2.658, 95%CI 1.770, 3.990) (Model 3, Table 2). Those who found one of the misinformation statements – “The COVID-19 vaccine is part of a big business plan by pharmaceutical companies” – true had significantly lower odds of intention to get vaccinated in the next month (OR = 0.712, 95%CI 0.513, 0.990) (Model 3, Table 2).

**Table 2:** Logistic regression analysis predicting vaccination intention in the next month

	Model 1		Model 2		Model 3	
	OR	95% CI	OR	95% CI	OR	95% CI
White	0.266	(0.143, 0.494)	0.256	(0.127, 0.516)	0.296	(0.140, 0.627)
≥50,000	0.584	(0.358, 0.951)	0.555	(0.302, 1.019)	0.686	(0.369, 1.274)
Christian	0.316	(0.192, 0.520)	0.450	(0.246, 0.823)	0.410	(0.220, 0.767)
Clerical	1.122	(0.695, 1.811)	1.126	(0.632, 2.007)	1.371	(0.754, 2.492)
Female	0.618	(0.373, 1.023)	0.708	(0.403, 1.245)	0.674	(0.373, 1.221)
Non-Hispanic	0.673	(0.363, 1.249)	0.721	(0.361, 1.440)	0.781	(0.372, 1.643)
Age	0.887	(0.795, 0.989)	0.855	(0.744, 0.983)	0.893	(0.772, 1.032)
Pol. ideology	1.003	(0.843, 1.193)	1.173	(0.943, 1.459)	1.155	(0.923, 1.446)
Attitudes			1.391	(0.960, 2.016)	1.314	(0.897, 1.925)
Confidence			2.665	(1.829, 3.884)	2.658	(1.770, 3.990)
Col. resp			0.763	(0.546, 1.065)	0.775	(0.547, 1.099)
Calculation			0.991	(0.723, 1.358)	1.201	(0.860, 1.676)
Susceptibility			1.027	(0.730, 1.446)	1.041	(0.729, 1.486)
Severity			1.038	(0.775, 1.389)	1.140	(0.841, 1.545)
Benefits			1.160	(0.796, 1.689)	0.924	(0.613, 1.393)
Barriers			0.832	(0.615, 1.125)	1.164	(0.796, 1.702)
Misinfo 1					0.883	(0.626, 1.246)
Misinfo 2					0.755	(0.550, 1.036)
Misinfo 3					0.921	(0.650, 1.307)
Misinfo 4					0.938	(0.678, 1.297)
Misinfo 5					0.842	(0.612, 1.156)
Misinfo 6					0.712	(0.513, 0.990)
Misinfo 7					1.038	(0.734, 1.469)

Notes: Bolded lines indicate that the predictor is significantly associated with the outcome

*Pol. Ideology* Political ideology, *Col. resp* Collective responsibility

Misinfo 1: The COVID-19 vaccine is not safe because it was rapidly developed and tested

Misinfo 2: Individuals who have already had COVID-19 and recovered don’t need to get a COVID-19 vaccine

Misinfo 3: COVID-19 vaccines cause infertility (i.e., unable to have children) or miscarriage

Misinfo 4: The COVID-19 vaccines don’t work because you can still get COVID after vaccination

Misinfo 5: The vaccine will alter my DNA

Misinfo 6: The COVID-19 vaccine is part of a big business plan by pharmaceutical companies

Misinfo 7: The COVID-19 vaccines don’t work because you can still be hospitalized due to COVID or even die from it

For individuals’ intention to receive COVID-19 vaccines in the next three to five years, those with household income higher than $50,000 (vs. <$50,000) (OR = 0.327, 95% CI 0.170, 0.627) and non-Hispanics (vs. Hispanics) (OR = 0.170, 95% CI 0.073, 0.397) had significantly lower odds of intention to get vaccinated in the next three to five years. Those with clerical, office, or sales jobs (vs. other types of jobs) (OR = 1.911, 95% CI 1.042, 3.504) had significantly higher odds of intention to get vaccinated in the next three to five years (Model 3, Table 3). With regards to independent variables, more positive attitudes (OR = 1.439, 95% CI 1.024, 2.024), stronger confidence in COVID-19 vaccines (OR = 1.568, 95% CI 1.105, 2.226), stronger preference in calculating the benefits of COVID-19 vaccines (OR = 2.108, 95% CI 1.541, 2.882), greater perceived susceptibility (OR = 1.471, 95% CI 1.045, 2.070) and severity (OR = 1.362, 95% CI 1.020, 1.820) were significantly associated with higher odds of intention to get vaccinated in the next three to five years (Model 3, Table 3). In contrast, a stronger sense of collective responsibility for receiving COVID-19 vaccines (OR = 0.604, 95% CI 0.426, 0.856) was significantly associated with lower odds of intention to get vaccinated in the next three to five years (Model 3, Table 3). None of the misinformation statements were associated intention to get vaccinated in the next three to five years.

**Table 3:** Logistic regression analysis predicting vaccination intention in the next three to five years

	Model 1		Model 2		Model 3	
	OR	95% CI	OR	95% CI	OR	95% CI
White	0.556	(0.295, 1.049)	0.844	(0.386, 1.843)	0.859	(0.385, 1.914)
≥50,000	0.278	(0.172, 0.450)	0.320	(0.168, 0.607)	0.327	(0.170, 0.627)
Christian	0.960	(0.601, 1.533)	1.264	(0.690, 2.316)	1.255	(0.676, 2.332)
Clerical	2.368	(1.497, 3.746)	1.898	(1.045, 3.447)	1.911	(1.042, 3.504)
Female	0.491	(0.313, 0.771)	0.686	(0.398, 1.183)	0.724	(0.413, 1.269)
Non-Hispanic	0.228	(0.119, 0.435)	0.158	(0.069, 0.362)	0.170	(0.073, 0.397)
Age	1.118	(1.012, 1.236)	0.992	(0.864, 1.139)	0.992	(0.863, 1.141)
Pol. ideology	0.817	(0.686, 0.971)	0.981	(0.785, 1.227)	0.976	(0.776, 1.227)
Attitudes			1.417	(1.018, 1.973)	1.439	(1.024, 2.024)
Confidence			1.584	(1.125, 2.230)	1.568	(1.105, 2.226)
Col. resp			0.607	(0.431, 0.857)	0.604	(0.426, 0.856)
Calculation			2.095	(1.557, 2.818)	2.108	(1.541, 2.882)
Susceptibility			1.396	(1.007, 1.935)	1.471	(1.045, 2.070)
Severity			1.330	(1.006, 1.760)	1.362	(1.020, 1.820)
Benefits			1.415	(0.970, 2.062)	1.365	(0.922, 2.020)
Barriers			0.716	(0.506, 1.012)	0.724	(0.501, 1.046)
Misinfo 1					0.843	(0.599, 1.187)
Misinfo 2					0.897	(0.661, 1.216)
Misinfo 3					0.981	(0.704, 1.366)
Misinfo 4					1.043	(0.751, 1.447)
Misinfo 5					1.113	(0.810, 1.529)
Misinfo 6					0.922	(0.674, 1.262)
Misinfo 7					1.089	(0.774, 1.532)

Notes: Bolded lines indicate that the predictor is significantly associated with the outcome

*Pol. Ideology* Political ideology, *Col. resp* Collective responsibility

Misinfo 1: The COVID-19 vaccine is not safe because it was rapidly developed and tested

Misinfo 2: Individuals who have already had COVID-19 and recovered don’t need to get a COVID-19 vaccine

Misinfo 3: COVID-19 vaccines cause infertility (i.e., unable to have children) or miscarriage

Misinfo 4: The COVID-19 vaccines don’t work because you can still get COVID after vaccination

Misinfo 5: The vaccine will alter my DNA

Misinfo 6: The COVID-19 vaccine is part of a big business plan by pharmaceutical companies

Misinfo 7: The COVID-19 vaccines don’t work because you can still be hospitalized due to COVID or even die from it

## Study 2 discussion

The main objective of Study 2 was to identify predictors of individuals’ intentions to receive COVID-19 vaccines in the next month and in the next three to five years. Stronger confidence in COVID-19 vaccines was consistently associated with higher odds of intentions to get vaccinated in the next month and in the next three to five years. This echoes the uncertainty shared by participants in Study 1, indicating that the unvaccinated would likely change their minds once they have more confidence in the safety and efficacy of COVID-19 vaccines.

Despite the consistent role of confidence in vaccination intentions across different timepoints, other findings indicate that short-term vaccination intention might be distinctively different from long-term vaccination intention. For instance, household income was not significantly associated with vaccination intention in the next month, while those who make higher than $50,000 (vs. those who make less than $50,000) were associated with lower odds of getting vaccinated in the next three to five years. Similarly, ethnicity was not significantly associated with vaccination intention in the next month, while non-Hispanics (vs. Hispanics) showed lower odds of intention to get vaccinated in the next three to five years.

A more interesting pattern emerged when independent variables were introduced in the logistic regression models. Rational evaluations on COVID-19 vaccines or contracting COVID-19 were associated with higher odds of intentions to get vaccinated in the next three to five years, although they were not significantly associated with intentions to get vaccinated in the next month. This advances literatures on vaccine hesitancy involving HBM and 5C [43–47], as little research has differentiated the relationships between predictors and vaccination intentions with different timepoints.

Those who believed that the COVID-19 vaccines were a part of pharmaceutical companies’ business plans showed lower odds of vaccination intention in the next month, yet none of the misinformation statements were associated with long-term vaccination intention. This is consistent with previous findings, indicating that individuals remain unwilling to receive the COVID-19 vaccines partly due to misinformation [48–51]. Interestingly, the finding suggests that the role of misinformation in vaccine hesitancy might only be short-term.

Surprisingly, a stronger sense of collective responsibility was associated with lower odds of intention to get vaccinated in the next three to five years, which is not consistent with previous literatures supporting the role of altruistic motives in vaccine uptakes [31, 52]. Finally, political ideology was not found to be a significant predictor of two intention outcomes, despite the strong connection between individuals’ political ideology and COVID-19 vaccine issues [18, 53–55]. This might have something to do with the relatively young ages of survey participants. As noted earlier, a majority of participants (*n* = 389, 80.3%) considered themselves liberal. This would have caused political ideology not to be variant to a great degree. This finding suggests that political ideology might not be a reliable indicator of vaccine uptake at least among young adults.

## Overall discussion

### Principal results

We conducted focus groups and in-depth interviews (Study 1) and an online survey (Study 2) by recruiting unvaccinated 19-to-29-year-old individuals residing in Grand Forks County, North Dakota between February 2022 and April 2022. The overall goals of this research were to understand the ways in which receiving COVID-19 vaccines is construed and to compare the predictors of short-term vaccination intention (i.e., next month) with those of long-term vaccination intention (i.e., three to five years later).

The findings of Study 1 indicate that most unvaccinated participants are hesitant individuals, or “fence-sitters,” [56] except for a few anti-vaxxers. They tend to have a cautious “wait-and-see” attitude because of unforeseen risks and uncertainty involved with COVID-19 vaccines [57]. A major barrier for these hesitant young adults to receiving COVID-19 vaccines is related to the safety and efficacy of vaccines, fueled by fast clinical trials, emergency approval processes, and the vaccine distribution process involving monetary incentives. Future communication by local public health organizations should seek to educate young people about vaccines, what they do, how they are developed, and what to expect. Additionally, more public attention should be directed to long-term COVID symptoms to warn young people of the potential dangers that could occur without vaccination. Not surprisingly, strong confidence in COVID-19 vaccines increased the odds of both short-term and long-term vaccination intentions in Study 2. Yet, short-term and long-term vaccination intentions were construed differently. Misinformation was associated with short-term vaccination intention only, while individuals’ rational evaluations, such as attitudes toward receiving COVID-19 vaccines and perceived susceptibility to and severity of contracting COVID-19, were significantly associated with long-term vaccination intention only.

### Implications

A number of prior observational research showed that the lack of confidence in COVID-19 vaccines [45], adverse effects [58], and misinformation [48, 49, 59, 60] contributed to vaccine hesitancy, while perceived severity [46] of and susceptibility [61] to COVID-19, and a greater sense of collective responsibility [31] increased willingness to be vaccinated. It is worth noting that most of these findings were documented to predict vaccination intention among general adults in the early stage of vaccine rollout, except for only a few studies that examined unvaccinated individuals [33, 58]. Focusing on unvaccinated young adults in their 20s 16 months after the initial vaccine rollout, this study advances the literature on COVID-19 vaccine hesitancy.

Interestingly, unlike previous studies [31, 52], a greater sense of collective responsibility was associated lower odds of receiving COVID-19 vaccines in the next three to five years in Study 2. This might reflect cultural differences as the positive relationship between collective responsibility and vaccination intention was documented in Asian [31] and Arabic [52] countries. Furthermore, misinformation was associated with lower odds of short-term vaccination intention, but it was not associated with long-term vaccination intention. According to the construal level theory (CLT), near-future behaviors are influenced by situational characteristics, whereas distant-future behaviors are guided by abstract features, such as attitudes [62, 63]. The findings from Study 2 are consistent with the CLT in that misinformation generated by the evolving situation of COVID-19 has a more significant impact on short-term vaccination intention.

In response to COVID-19 vaccine hesitancy among young adults, a recent randomized controlled trial in Canada tested the effects of video intervention (vs. text intervention) featuring altruistic motives on vaccination intention among unvaccinated Canadian adults who are 20-to-39-years-old [64]. The study did not find a significant difference between video and text interventions in vaccination intention. Similarly, an online clinical trial conducted in Japan also found that messages comparing one’s vaccination decisions with others did not influence young adults’ vaccination intention [65]. These findings suggest that relying on collective responsibility, altruistic motives, or social norms might not effectively address vaccine hesitancy among young adults. The findings of Study 1 and Study 2, however, indicate that vaccine interventions must determine the behavioral goal first, given that immediate or gradual increase in vaccine uptake are different behaviors sharing a few similarities only. Given that this study investigated vaccine hesitancy among young people, more consistent education should be conducted about vaccines and about the importance of immunity in public health. Communicating about vaccines only in the midst of the pandemic is not sufficient for this age group, and public health officials should plan more educational communication about vaccines.

Based on the findings of Study 1, most of unvaccinated individuals are still weighing the benefits and risks of receiving COVID-19 vaccines. For them, the risks of vaccination are still much greater than potential harm from the disease. Intervention efforts, therefore, should consistently focus on communicating the safety and efficacy of COVID-19 vaccines at multiple levels of influence [59]. Consistent, targeted messaging should be conducted on platforms most frequently used by young people, including social and digital media [66]. This should contribute to both immediate and gradual increases in vaccine uptake. For a long-term perspective, vaccine promotions are recommended to be paired with describing how susceptible individuals are to contracting COVID-19 and how severe infections can be. According to the HBM [67], performing a recommended behavior, which is to receive COVID-19 vaccines, is unlikely to occur when individuals do not see COVID-19 as a viable threat.

Over the course of the COVID-19 pandemic, it has become clear that vaccine promotions should not take the “one-size-fit-all” approach [68]. Diverse demographic groups, such as racial/ethnic minorities [69–71] and LGBTQIA community members [72], have received empirical attention, but a focus on various geographical segments is somewhat limited. In this light, this study builds up on previous research providing a unique regional perspective [73–75].

### Limitations

This study has several limitations. First, both Study 1 and Study 2 used a convenience sampling recruited from social media and online promotions. This may limit the generalizability of findings. Second, the findings of both Study 1 and Study 2 might not be applicable to other regions in the U.S. or worldwide. Finally, the findings of both Study 1 and Study 2 do not have any empirical data to suggest how to communicate with this audience group; rather, the findings inform what to communicate when targeting hesitant young adults.

### Conclusion

This study identified several barriers to receiving COVID-19 vaccines by conducting a series of focus groups and in-depth interviews. Particularly, participants perceived there to be financial interest by both the CDC and the pharmaceutical drug companies. There was also a fear of the unknown related to the vaccines (e.g., infertility), and/or the virus itself, (e.g., long-term COVID). Through an online survey, this study also uncovered that stronger confidence in COVID-19 vaccines would play an important role in unvaccinated individuals’ intention to get vaccinated in the near and distant future. The strength of this study lies that the predictors of individuals’ intention to receive COVID-19 vaccines in the near future (i.e., in the next month) are vastly different from those of distant-future intention (i.e., three to five years later). This shows that there is a window of opportunity for health promotion and education to influence young people’s vaccine behaviors. To increase the vaccination rates among individuals in their 20s in the short-term, it is important to communicate the safety and efficacy information about the vaccines and address any concerns about profits and monetization associated with COVID-19 vaccines. Future research should be conducted to better understand how young people make decisions about COVID vaccines following promotional messaging. Given that there is a chance the world must “live” with COVID-19 for a longer period of time, it is recommended for public health and communication professionals to continue to communicate with hesitant young adults so that they can acknowledge that benefits of receiving COVID-19 vaccines outweighs the risks; and they can evaluate how susceptible they are to contracting COVID-19 as well as how severe infections can be.

## Data Availability

The datasets used and/or analyzed during the current study are available from the corresponding author on reasonable request. The dataset is not submitted at this time of writing, as the authors are conducting additional analyses. Informed consent procedure was approved by the ethics committee of University of North Dakota as data was collected anonymously.

## Abbreviations

5C: Five psychological antecedents of vaccination
CDC: Centers for Disease Control and Prevention
CI: Confidence intervals
CLT: Construal Level Theory
FDA: Food and Drug Administration
HBM: Health Belief Model
IRB: Institutional Review Board
LGBTQIA: Lesbian, Gay, Bisexual, Transgender, Queer, Intersex, Asexual
mRNA: Messenger RNA
OR: Odds ratio
SPSS: Statistical Package for the Social Sciences
WHO: World Health Organization
